# Common Periodontal Diseases of Children and Adolescents

**DOI:** 10.1155/2014/850674

**Published:** 2014-06-26

**Authors:** Hayat Al-Ghutaimel, Hisham Riba, Salem Al-Kahtani, Saad Al-Duhaimi

**Affiliations:** Department of Pediatric Dentistry, King Saud Bin Abdulaziz University for Health Science, Riyadh, Saudi Arabia

## Abstract

*Background.* Since 2000, studies, experiments, and clinical observations revealed high prevalence of periodontal diseases among children and adolescents. Therefore, this paper was designed to provide an update for dental practitioners on epidemiology, microbiology, pathology, prevention, diagnosis, and treatment of periodontal diseases in children and adolescents. *Methods.* This paper reviews the current literature concerning periodontal diseases in pediatric dentistry. It includes MEDLINE database search using key terms: “periodontal diseases in children,” “Periodontal diseasesin adolescents,” “periodontal diseases risk factors,” “microbiology of periodontal diseases,” “classification of periodontal diseases,” “epidemiology of periodontal diseases,” and “treatment of periodontal diseases.” Articles were evaluated by title and/or abstract and relevance to pediatric dentistry. Sixty-five citations were selected by this method and by the references within the chosen articles. A review of the comprehensive textbooks on pediatric dentistry and periodontology was done. Some recommendations were based on the opinions of experienced researchers and clinicians, when data were inconclusive.

## 1. Periodontium of the Primary Dentition

In medical dictionaries, the word periodontium comes from the Greek terms peri-, which means “around,” and -odons, which means “tooth.” Literally, it means that which is around the tooth. Periodontium includes the tissues that surround and support the teeth. Those tissues are gingiva, cementum, periodontal ligaments, and alveolar bone [1, 2]. A long time ago, it has been found that periodontium of the primary dentition differs from that of the permanent dentition in several aspects [3]. The gingiva in primary dentition appears to be more reddish, vascular, and flabby and to lack stippling [1, 4]. And the periodontal ligaments in children are wider and have less dense fibers [1, 3, 4]. The alveolar bone in primary dentition has less trabecula and calcification, more marrow spaces, and greater blood supply and lymphatic drainage [1, 3, 4]. At the molecular level, some investigators reported that periodontium of the primary dentition resorbed more easily because it contains more sialoprotein and osteoprotein, which facilitate the binding of odontoclast [1, 5–7]. 

## 2. Periodontal Diseases

### 2.1. Definition

Periodontal diseases constitute a group of conditions that are considered nowadays ubiquitous among children, adolescents, and adults [3]. The term “periodontal diseases” includes any inherited or acquired disorders of the tissues that are investing and supporting the teeth (gingiva, cementum, PDL, and alveolar bone) [2]. Another researcher defined periodontal diseases as chronic infectious disorders caused primarily by bacteria [14].

### 2.2. Epidemiology

In 1996, Albandar et al. assessed the prevalence of gingivitis among large group of adolescents in the United States and found that 82.1% of the participating subjects were having gingivitis [15]. Similar findings of high prevalence of gingivitis among children and adolescents were reported by other studies worldwide [16, 17]. Albandar et al., in another study, assessed the prevalence of early-onset forms of periodontitis among group of US adolescents and reported that 0.6% of the subjects were having juvenile periodontitis at the age of 13–15, and 2.75% of the subjects were having chronic periodontitis at the age of 16-17 [18]. Low prevalence of periodontitis among children and adolescents was reported by other studies in different populations [19]. Many researchers have observed larger amount of plaque and less inflammation in relation to the amount of plaque in children compared to the adults [3, 4, 14]. Furthermore, experts and clinicians noted that most of the periodontal diseases that affect children and adolescents are reversible and cause little tissue damage compared to the adults [4].

### 2.3. Causes

Periodontal diseases are most commonly caused by pathogenic microorganism in the oral biofilm or dental plaque that accumulated around the teeth due to poor oral hygiene [3, 4]. The evidences indicate that periodontal diseases develop when the numbers of Gram-negative bacteria and anaerobes in subgingival plaque increased [20, 21]. Numerous research efforts were implemented in order to identify bacterial species that are associated with the periodontal diseases [22, 23]. The most common periodontal-diseases-associated microorganisms were* Aggregatibacter* (*Actinobacillus*),* Porphyromonas gingivalis*,* Tannerella forsythensis*, and spirochaete* Treponema denticola* [2, 24–28]. Recent studies implicate fungi, such as* Candida albicans*, and Herpes viruses in the pathogenesis of periodontal diseases among immune-compromised children [29–31]. However, genetic, developmental, traumatic, neoplastic, and metabolic factors contributed to the cause of these diseases [9, 11, 24]. Furthermore, some systemic diseases and medications also have periodontal manifestations [2–4].

### 2.4. Classification

**Figure 1:**
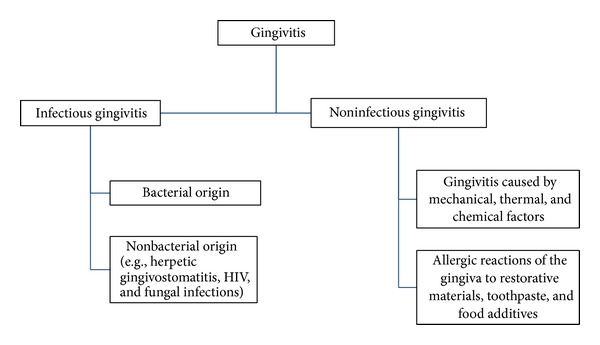
Classification of gingivitis.

Over the last few decades, the nomenclature and classification of periodontal diseases changed periodically [3]. Regardless of the causative factors, periodontal diseases are divided into destructive and nondestructive form [14]. Gingivitis is a reversible and nondestructive form of periodontal diseases [14, 32, 33]. It is the inflammation of the marginal gingiva that may progress to include free and attached gingiva but causing no attachment loss [9, 11]. Based on clinical findings and diagnosis, gingivitis was subdivided into infectious and noninfectious forms as in Figure 1 [14, 34–37]. On the other hand, the irreversible and destructive form of periodontal diseases is periodontitis [14]. It is the inflammation of the tooth supporting tissue, which is accompanied by loss of connective tissue attachment and breakdown of the supporting alveolar bone [9, 11]. Periodontitis may progress to cause exposure of the roots, mobility, and premature loss of the teeth [9]. In 1989, the American Academy of Periodontology set criteria in order to distinguish various forms of periodontitis [3]. Those criteria are (1) age at onset, (2) distribution of the sites affected by the disease, (3) presence or absence of the systemic diseases, (4) rate of the disease progression, (5) response to treatment, and (6) presence or absence of specific host or microbial factors (the consensus of the world workshop in clinical periodontics) [3]. The most recent classification of periodontal diseases was introduced in 1999 by international workshop of periodontology and includes greater variety of periodontal diseases categories [3, 38, 39]. However, this paper will not follow specific classification system but rather will focus primarily on the periodontal diseases that are most commonly seen in pediatric dental patients.

#### 2.4.1. Gingivitis

As mentioned earlier in this paper, gingival problems, either in acute or chronic nature, are nearly universal among children and adolescents [19]. Diagnosis of various types of gingivitis relied mainly on the clinical findings and manifestations [3, 32]. Those findings include redness and edema of the marginal gingiva and bleeding upon probing [2–4]. As disease persists, gingival margin may become rolled, interdental papilla may become enlarged and bulbous, bleeding may start spontaneously, and probing depth may increase as a consequence of gingival overgrowth (hyperplasia and hypertrophy) [2, 3, 32, 34].

Histologically, ulceration of the sulcular epithelium was observed both in children and in adolescents [32, 34]. However, researchers have noted predominance of T-lymphocyte infiltrate in gingivitis in children compared to B-cell (plasma cells) infiltrate in gingivitis in adolescents (Ranney et al., 1981, and Page and Schroeder, 1976) [3, 4]. Although the microbiological picture of gingivitis in children and adolescents has not been completely characterized, certain bacterial species have been found in experimental studies [4]. Those species were* Aggregatibacter (Actinobacillus) *sp.,* Capnocytophaga *sp.,* Leptotrichia* sp., and* Selenomonas* sp. [27, 28].

Gingival problems that are commonly seen in children and adolescents are as follows.

(*1) Eruption Gingivitis. *Some gingival inflammation normally accompanies eruption process [4, 9, 11]. Poor oral hygiene by neglect or as a consequence of malalignment of the erupting teeth will aggravate gingival inflammation [2–4, 9, 11]. Usually, the condition will subside as the oral hygiene improves and the tooth reaches normal occlusion [2, 4, 9, 11]. Plaque control regimen is the treatment of eruption gingivitis [4]. 

(*2) Pubertal Gingivitis. *Pubertal gingivitiswhich isalso called steroid hormone-related gingivitis [3] is defined as exacerbation of gingivitis by fluctuation in gonadotrophic hormone levels during puberty [3, 4, 11]. A similar condition is seen during pregnancy (Loe, 1965) and in females taking contraceptives (Kalkwarf, 1978) [2–4, 11]. The phenomenon of this condition can be explained as any increase in the levels of estrogen and progesterone in the gingival tissues resulting in vasodilatation and proliferation, increase in gingival vascularity, and increase in susceptibility of inflammation in the presence of local factors [4, 11, 40, 41].

Pubertal gingivitis is characterized by swelling of the interdental papilla, with spontaneous gingival hemorrhage [3, 4]. Professional prophylaxis and removal of the local factors combined with good oral hygiene regimen at home will result in major improvements [4]. In some cases, gingival swelling becomes fibrotic and necessitates surgical excision in the future [3].

(*3) Gingivitis Related to Mouth-Breathing. *Mouth-breathing causes desiccation of the oral tissue and consequently gingival inflammation and halitosis [4, 11]. Immediate management for the problem includes (1) maintaining good oral hygiene, (2) lubrication of the tissue, and (3) the use of the oral screen to cover the tissue during sleeping [4]. Elimination of the problem requires comprehensive treatment plan by an orthodontist and an otolaryngologist [3, 26].

(*4) Drug Induced Gingival Overgrowth. *Certain classes of medications have been approved to cause gingival overgrowth and aggravate gingival inflammation in the presence of local factors [3, 4, 41]. Those medications are cyclosporine (immune-depressant), phenytoin (anticonvulsant), and calcium channel blockers (antihypertensive) [2, 3, 11, 41]. Gingival overgrowth was noted in 30% of patients taking cyclosporine, 50% of patients using phenytoin, and 15% of patients medicated with calcium channel blockers such as nifedipine, verapamil, and amlodipine [3, 41, 42]. This kind of gingival overgrowth usually starts at the interdental area and then spreads to include marginal gingiva [3, 4, 41]. Occasionally, it can be so severe to cover the incisal and occlusal surfaces of the teeth [11, 41]. However, its severity is closely related to the amount of accumulated plaque [4, 11, 41].

The pathogenesis of this condition is uncertain yet [11]. However, the interaction between those drugs and/or metabolites and fibroblast will lead to fibroepithelial gingival overgrowth, epithelial acanthosis, increase in fibroblast number, and increase in collagen production [43].

The management of this condition starts from improving patient's oral hygiene by both mechanical and chemical plaque control techniques [4, 11]. In addition, professional scaling and polishing are required to remove all the local aggravating factors [2–4, 11]. Sometimes, gingivectomy and gingivoplasty are needed for gingival recontouring in order to improve esthetic and hygiene [3, 4]. Dentist should not try to stop or replace patient medications [4]. However, a consultation with the patient's physician can be done to determine the possibility of drug replacement [3, 44].

(*5) Gingivitis Associated with Malnutrition. *There is strong evidence that hypovitaminoses and mineral deficiency associated with specific manifestation in oral and perioral area may lead to periodontal diseases [2, 11]. For example, vitamin C deficiency will cause scurvy, which is manifested as a decrease in the production and maintenance of collagen [11]. Oral scurvy is characterized by painful gingival swelling, gingival edema, and hemorrhage on slight provocation [11, 45]. “Scorbutic gingivitis” results when severe vitamin C deficiency is combined with poor oral hygiene [11, 46, 47]. However, it is characterized by ulcerative gingivitis, fetid odor, rapid development of periodontal pocket, and tooth loss [46, 47].

(*6) Acute Necrotizing Ulcerative Gingivitis (ANUG). *Trench mouth or Vincent's infection is an acute gingival inflammation caused mainly by a special bacterial species called* Borrelia vincentii* [4, 9, 11]. Occasionally, other anaerobes and spirochetes such as* Fusobacterium* spp.,* Selenomonas* spp.,* Prevotella* spp., and* Treponema* spp. are observed in microbiological culture [48]. The risk factors include poor oral hygiene, stress, decreased host resistance, and HIV infection [4, 11]. ANUG is characterized by punched-out interdental papilla that is covered with a grayish-white pseudomembrane, which may extend to cover marginal gingiva [4, 48, 49]. Patients are usually suffering from strong continuous pain and fetid odor as a result of bacterial reaction's end products, bacterial toxins, and tissue necrosis [4, 49]. Generalized systemic manifestation including low-grade fever, lymphadenopathy, and malaise is often accompanying ANUG [48, 49].

Both local and systemic therapy are needed for the treatment of ANUG [4, 48, 49]. The first step is professional gentle scaling to remove local deposits as well as necrotic tissue [4]. Patients are instructed to follow strict daily oral hygiene regimen [4, 49]. Oxidizing mouthwash such as chlorhexidine may help to restore microbial balance [4, 48, 49]. 250–500 mg per dose of penicillin or erythromycin was recommended for five days [4, 48, 49]. Flagyl (metronidazole) is approved by evidence to help in eliminating the acute symptoms rapidly [11].

(*7) Primary Herpetic Gingivostomatitis. *Primary herpetic gingivostomatitis is defined as an acute gingival condition that is caused by Herpes simplex virus type I [3, 4]. Its clinical picture is characterized by a painful gingival inflammation and vesicles that are formed mainly on the dorsum of the tongue, hard palate, and gingiva [4]. Those vesicles ruptured eventually, leaving a painful ulcer with a yellow gray floor and red halo [3, 4, 11]. Lymphadenopathy, fever, and malaise are common systemic features for herpetic gingivostomatitis [48, 49]. It is commonly affecting children under the age of ten with a peak incidence at 2–4 years of age [4]. The condition is self-limiting and required symptomatic treatment only [3, 4]. However, systemic antiviral therapy is needed in immunocompromised patients [11].

#### 2.4.2. Periodontitis

(*1) Chronic Periodontitis (Incipient). *Although this form of periodontitis is considered more prevalent in adults, it can be seen occasionally in children and adolescents [9]. Comparing to aggressive periodontitis, chronic periodontitis is characterized by a low to moderate rate of progression that may include episodes of rapid destruction [9, 11]. It is subdivided according to the percentage of the involved sites into localized (<30%) and generalized (>30%) [2, 3, 9]. Furthermore, it can be subdivided according to the severity of the disease into mild (1-2 CAL), moderate (3-4 CAL), and severe (≥5 CAL) [9, 11].

(*2) Aggressive Periodontitis.* Aggressive periodontitis which is also called “juvenile periodontitis” is considered to be prevalent in children and adolescents during circumpubertal period [3, 10, 11]. It is characterized by rapid loss of connective tissue attachment and alveolar bone with familial aggregation [11]. It is caused by both pathogenic microflora and abnormality in host defense mechanisms [3]. Aggressive periodontitis can be subdivided into localized (LAgP) and generalized form (GAgP).

Localized aggressive periodontitis patients have interproximal attachment loss on no more than two teeth other than first permanent molars and incisors [3, 10, 11]. At the microbiological level, up to date, no single species of microorganism has been found in all cases of LAgP [9, 50]. However,* Aggregatibacter* (*Actinobacillus*) sp. in combination with* Bacteroides*-like sp. and* Eubacterium* sp. has been isolated from most of LAgP cases [51–54]. It is well documented that LAgP is associated with a variety of functional defects in neutrophils [10, 55].

Generalized aggressive periodontitis patients have interproximal attachment loss on at least three teeth that are not permanent first molars or incisors [3, 10]. It is usually affecting the entire dentition and is considered as a disease of adolescents and young adults [10]. At the microbiological level,* Porphyromonas gingivalis* and* Treponema denticola* were isolated from most GAgP cases [3, 10]. Patients with GAgP have defective neutrophil functions and reduction in GP-110 [10, 56]. Furthermore, alteration in IgG was reported to be present in both forms of aggressive periodontitis [10]. IgG is known to have a protective and disease-limiting effect [10].

The successful treatment of aggressive periodontitis includes surgical or nonsurgical periodontal therapy in combination with systemic antibiotic therapy [10, 57]. According to the number of studies, the most successful antibiotic in the treatment of aggressive periodontitis is tetracycline alone or with metronidazole [10, 57–59], followed by metronidazole in combination with amoxicillin in the presence of tetracycline resistance [10, 60].

(*3) Periodontitis as a Manifestation of Systemic and Genetic Disorders. *Include a group of rare diseases that predispose the affected individual to highly destructive periodontal infections [8, 9, 12]. Those diseases were characterized by defective functions of neutrophils and/or other immune cells [9]. The most common systemic diseases and genetic disorders that are associated with periodontal conditions are listed in Table 1.

The treatment of periodontitis as a manifestation of systemic diseases includes a combination of surgical and nonsurgical therapy in addition to antibiotic therapy [8, 9, 61, 62]. However, the success of treatment of periodontitis as a manifestation of systemic diseases is considered unpredictable [8, 9].

## Figures and Tables

**Table 1:** Systemic and genetic disorders associated with periodontal diseases in children and adolescents.

Systemic or genetic disorder	Nature of the disorder	Periodontal and other manifestations
Insulin dependent diabetes mellitus (IDDM)	Decrease in insulin secretion or availability caused by genetic defect in pancreatic beta-cells [8–10].	(i) Gingivitis, attachment loss, and bone loss are more prevalent in poorly controlled cases [4]. (ii) Reduced PMNs functions (chemotaxis, adhesion, and phagocytosis) [3, 11]. (iii) Decreased collagen synthesis and increased collagenase activity [4]. (iv) Delayed wound healing [3, 4]. (v) Increased susceptibility to infections [8–10].

HIV/AIDS	HIV/AIDS develops as a result of infection with human immunodeficiency virus [3].	(i) Linear gingival erythema [3, 4]. (ii) Acute necrotizing ulcerative gingivitis [3, 4, 11]. (iii) Acute necrotizing periodontitis [12, 13].

Leukocyte adhesion deficiency (LAD)	Inherited as autosomal recessive condition in which glycoprotein adhesion in leukocyte molecules is severely reduced [3, 11].	(i) Poor immune response to bacterial infections [3, 4]. (ii) Acute inflammation and rapid bone loss [3, 4, 11]. (iii) Recurrent bacterial infections [3]. (iv) Poor wound healing [3, 4]. (v) Associated with prepubertal periodontitis [3, 8, 11].

Leukemia	Uncontrolled proliferation of white blood cells [3, 4].	(i) Gingival hyperplasia and hypertrophy [3, 4]. (ii) Gingival pallor [3, 4, 11]. (iii) Spontaneous gingival hemorrhage and petechiae [3, 8].

Neutropenia	The number of PMNs in peripheral blood is below 1000/mm^3^ in infants and 1500/mm^3^ in children [3, 4].	(i) Severe gingivitis, gingival ulcerations, and periodontitis [3, 4]. (ii) Recurrent infections such as otitis media and upper respiratory infections [3, 9, 11].

Acrodynia	Acrodynia is caused by mercurial toxicity reaction (mercury poisoning or idiosyncrasy to mercury) [3, 4, 11].	(i) Gingival and mucosal hyperplasia [3]. (ii) Alveolar bone loss [3, 4]. (iii) Early loss of primary teeth [3, 4]. (iv) Profuse salivation and sweating [3, 11].

Histiocytosis X	Disturbance of the reticuloendothelial system includes defects in PMNs and monocyte [3, 4, 11].	(i) Increased susceptibility to bacterial infections [11].

Hypophosphatasia	Genetic disorder characterized by low level of serum alkaline phosphatase and excretion of phosphoethanolamine in urine [3, 4, 11].	(i) Premature loss of deciduous teeth and skeletal deformity [3, 4, 11]. (ii) Defective bone/tooth mineralization [3, 4, 11]. (iii) Cementum hypoplasia/aplasia [3, 4, 11].

Chediak-Higashi syndrome	Autosomal recessive disorder characterized by impaired function of cytoplasmic microtubules in PMNs [3, 4, 11].	(i) Recurrent infections [3]. (ii) Severe gingivitis and periodontitis [4]. (iii) Intraoral ulcerations [3, 11].

Papillon-Lefevre syndrome	Autosomal recessive condition associated with impaired neutrophil functions [3, 4, 11].	(i) Palmoplantar hyperkeratosis [3]. (ii) Early-onset periodontitis affecting both primary dentition and permanent dentition [3].

Down syndrome	Trisomy 21, mongolism, and autosomal chromosomal anomaly associated with impaired PMNs functions, connective tissue disorders, and gingival hyperinnervation [3, 11].	(i) Gingivitis and periodontitis especially in lower anteriors [11]. (ii) Enamel hypoplasia [3, 4, 11]. (iii) Microdontia [3, 4, 11]. (iv) Macroglossia [3, 4, 11]. (v) Fissured tongue [3, 4, 11].

Ehlers-Danlos syndrome	Collage disorder affecting joints and skin. Ten type; type VIII is autosomal dominant and has periodontal implications [11].	(i) Aggressive early-onset periodontitis [11]. (ii) Prolonged bleeding [3]. (iii) Easily traumatized mucosa. [11]
